# Interrelationship among Dental, Skeletal and Chronological Ages in Urban and Rural Female Children

**DOI:** 10.5005/jp-journals-10005-1058

**Published:** 2010-08-17

**Authors:** K Chaudhry, A Agarwal, U Rehani

**Affiliations:** 1 Senior Lecturer, Department of Pedodontics and Preventive Dentistry, Uttaranchal Dental and Medical Research Institute Dehradun, Uttarakhand, India; 2 Reader, Department of Pedodontics and Preventive Dentistry, Subharti Dental College, Meerut, Uttar Pradesh, India; 3 Professor and Head, Department of Pedodontics and Preventive Dentistry, Subharti Dental College, Meerut, Uttar Pradesh, India

**Keywords:** Dental age, chronological age, skeletal age, Demirjian’s method.

## Abstract

***Aim:*** This study was an attempt, to determine chronological age, dental age and skeletal age, and to establish interrelationship, amongst the dental, skeletal and chronological ages and their differences if any between rural and urban female children. The study included 80 girls aged 8 to 14 years from rural and urban areas.

***Material and methods:*** The subjects were divided into 4 Groups: Group I, II, III, and IV. Group I and II comprising of rural female subjects, wherein Group I comprised of 8 to 11 years and Group II comprised of 11 to 14 years old females. Group III and IV comprising of urban females wherein Group III included 8 to 11 years and Group IV comprised of 11 to 14 years old females. Orthopantomograms and hand and wrist radiographs were taken. The calcification status of permanent teeth was evaluated from orthopantomograms, and dental age was calculated according to Demirjian’s method. The stages of ossification of various carpal bones were evaluated from the hand-wrist radiograph using radiographic atlas of Greulich and Pyle and skeletal age was calculated. The chronological age was recorded from the actual date of birth.

***Results:*** Data collected was statistically analyzed.

***Conclusion:*** Highly significant correlation was observed between dental and skeletal age (r=0.752, p-value < 0.01) in total sample. Strong correlation of chronological age with dental and skeletal age was also observed (r=0.650, r = 0.620, respectively). Out of all three correlations, dental age and skeletal age had the maximum correlation in total sample. While comparing rural and urban sample as regard to ages or correlations no significant difference was found (p-value < 0.01).

## INTRODUCTION

Age determination plays a great role in forensic science, pediatric endocrinology and is of particular interest in orthodontic and pedodontic treatment planning.^1^

The usefulness of the somatic and sexual, maturity indicators has limited value for the immediate clinical judgment of a patient’s maturity stage because these indicators can be applied only after the serial recording of height or the inception of puberty.

The technique for assessing skeletal maturity consists of visual inspection of the developing bones, their initial appearance and their subsequent ossification-related changes in shape and size. Various areas of the skeleton have been used: the foot, the ankle, the hip, the elbow, the hand-wrist, and the cervical vertebrae.^2^ The hand-wrist radiograph is commonly used for skeletal developmental assessment. One of the most frequently applied method to estimate skeletal age is the atlas of Greulich and Pyle.^3^

Dental age is of particular interest to the pedodontist and orthodontist in the management of different types of malocclusions in relation to maxillofacial growth.^1^

Demirjian^7^ et al formulated the method of dental age assessment by reference to the radiological appearances of the seven teeth on the left side of the mandible.

Relations between the dental and skeletal ages have been evaluated in order to correlate the two ages for purposes of diagnosis.

These parameters are however, influenced by differences in the life-styles, dietary patterns and socioeconomic status.^4,5^

## METHODOLOGY

Eighty subjects of 8 to 14 years of age from various schools of rural and urban areas coming to Department of Pedo-dontist and Preventive Dentistry, Subharti Dental College Meerut, were randomly selected. The criteria for selection of cases for the present study was as follows:

- The subject should be clinically free from any disorder affecting growth.
- Subject should be clinically free from any past prolonged illness.
- Subject should had a complete mandibular permanent dentition (erupted or not).

The study was carried out in following steps:

- Brief history of the child was taken including child’s name, age, sex, date of birth, father’s name, address and school. Date of birth of each child was checked from school records.
- Dental examination was done with a probe, mouth mirror, and tweezer under good illumination and the state of eruption of teeth was seen.
- Informed consent was taken.
- Orthopantomograms were taken.
- Radiographs of hand and wrist of left side was taken on “8” × “10” X-rays films (Kodak) by seating the individual on an adjustable stool in front of the table, so that the subject could place the forearm on it along a line parallel to the shoulders. The cassette was then placed with its long axis parallel to the long axis of the hand and exposed for 0.04 seconds to X-radiation at 46 KVP and 100 MA.

Both the radiographs for each subject was taken in the Department of Oral Medicine and Radiology. All the exposed films were developed, fixed and dried.

The radiographs selected were clear, of good quality, with all the lower permanent mandibular teeth present on left side on orthopantomogram, and all the carpal bones clearly visible in the hand and wrist radiograph. The ortho-pantomograms were viewed on X-ray viewer. The state of calcification of seven left permanent mandibular teeth was seen and dental age was calculated according to Demirjian’s method and recorded on the proforma.

Hand and wrist radiographs were viewed on a view box and the state of ossification of various carpal bones were seen and recorded on the proforma.

The chronological age, dental age and skeletal age was calculated as follows:

### Dental Age Assessment: Demirjian’s Method

**Fig. 1:**
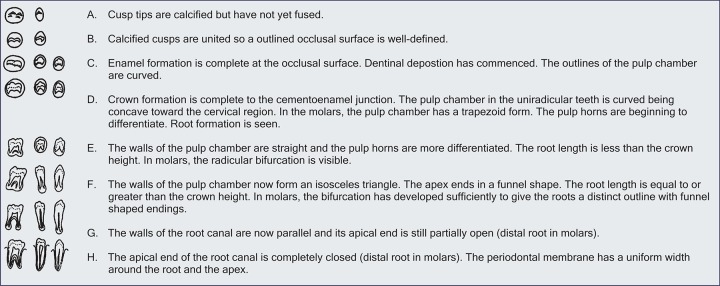
Tooth calcification stages from A to H

*Method:* A new method was given for estimating dental age, by reference to the radiological appearances of the seven teeth on the left side of the mandible. Eight stages, A to H, have been defined from the first appearance of calcified points to the closure of the apex (Fig. 1). The summed scores on all seven teeth give a dental maturity score which can be converted directly into a dental age, as shown in Tables 1 and 2.

### Chronological Age

Chronologic age of an individual was calculated by subtracting the birth date from the date on which the radiographs were exposed for that particular individual. Decimal age was taken for simplicity of statistical calculation and ages were estimated on yearly basis, e.g. 11 years 6 months as 11.5 years and it was included in 11 to 14 years age group.

**Table 1:** **Table 1:** Self-weighted scores for dental stages: Seven teeth (Mandibular left side)

		*Stage*																	
Tooth		0		A		B		C		D		E		F		G		H	
M_2_		0.0		2.7		3.9		6.9		11.1		13.5		14.2		14.5		15.6	
M_1_								0.0		4.5		6.2		9.0		14.0		16.2	
PM_2_		0.0		1.8		3.4		6.5		10.6		12.7		13.5		13.8		14.6	
PM_1_						0.0		3.7		7.5		11.8		13.1		13.4		14.1	
C								0.0		3.8		7.3		10.3		11.6		12.4	
I_2_								0.0		3.2		5.6		8.0		12.2		14.2	
I_1_										0.0		2.4		5.1		9.3		12.9	

NB: Stage 0 is no calcification.

**Table 2:** **Table 2:** Conversion of maturity score to dental age: Seven-teeth (Mandibular left side)

*Age*		*Score*		*Age*		*Score*		*Age*		*Score*		*Age*		*Score*	
3.0		13.7		7.0		51.0		11.0		94.5		15.0		99.2	
0.1		14.4		0.1		52.9		0.1		94.7		0.1		99.3	
0.2		15.1		0.2		55.5		0.2		94.9		0.2		99.4	
0.3		15.8		0.3		57.8		0.3		95.1		0.3		99.4	
0.4		16.6		0.4		61.0		0.4		95.3		0.4		99.5	
0.5		17.3		0.5		65.0		0.5		95.4		0.5		99.6	
0.6		18.0		0.6		68.0		0.6		95.6		0.6		99.6	
0.7		18.8		0.7		71.8		0.7		95.8		0.7		99.7	
0.8		19.5		0.8		75.0		0.8		96.0		0.8		99.8	
0.9		20.3		0.9		77.0		0.9		96.2		0.9		99.9	
4.0		21		8.0		78.8		12.0		96.3		16.0		100.0	
0.1		21.8		0.1		80.2		0.1		96.4					
0.2		22.5		0.2		81.2		0.2		96.5					
0.3		23.2		0.3		82.2		0.3		96.6					
0.4		24.0		0.4		83.1		0.4		96.7					
0.5		24.8		0.5		84.0		0.5		96.8					
0.6		25.6		0.6		84.8		0.6		96.9					
0.7		26.4		0.7		85.3		0.7		97.0					
0.8		27.2		0.8		86.1		0.8		97.1					
0.9		28.0		0.9		86.7		0.9		97.2					
5.0		28.9		9.0		87.2		13.0		97.3					
0.1		29.7		0.1		87.8		0.1		97.4					
0.2		30.5		0.2		88.3		0.2		97.5					
0.3		31.3		0.3		88.8		0.3		97.6					
0.4		32.1		0.4		89.3		0.4		97.7					
0.5		33.0		0.5		89.8		0.5		97.8					
0.6		34.0		0.6		90.2		0.6		98.0					
0.7		35.1		0.7		90.7		0.7		98.1					
0.8		36.8		0.8		91.1		0.8		98.2					
0.9		37.0		0.9		91.4		0.9		98.3					
6.0		38.0		10.0		91.8		14.0		98.3					
0.1		39.1		0.1		92.1		0.1		98.4					
0.2		40.2		0.2		92.3		0.2		98.5					
0.3		41.3		0.3		92.6		0.3		98.6					
0.4		42.5		0.4		92.9		0.4		98.7					
0.5		43.9		0.5		93.2		0.5		98.8					
0.6		45.2		0.6		93.5		0.6		98.9					
0.7		46.7		0.7		93.7		0.7		99.0					
0.8		48.0		0.8		94.0		0.8		99.1					
0.9		49.5		0.9		94.2		0.9		99.1					

### Skeletal Age Assessment: Greulich and Pyle Method

**Fig. 2:**
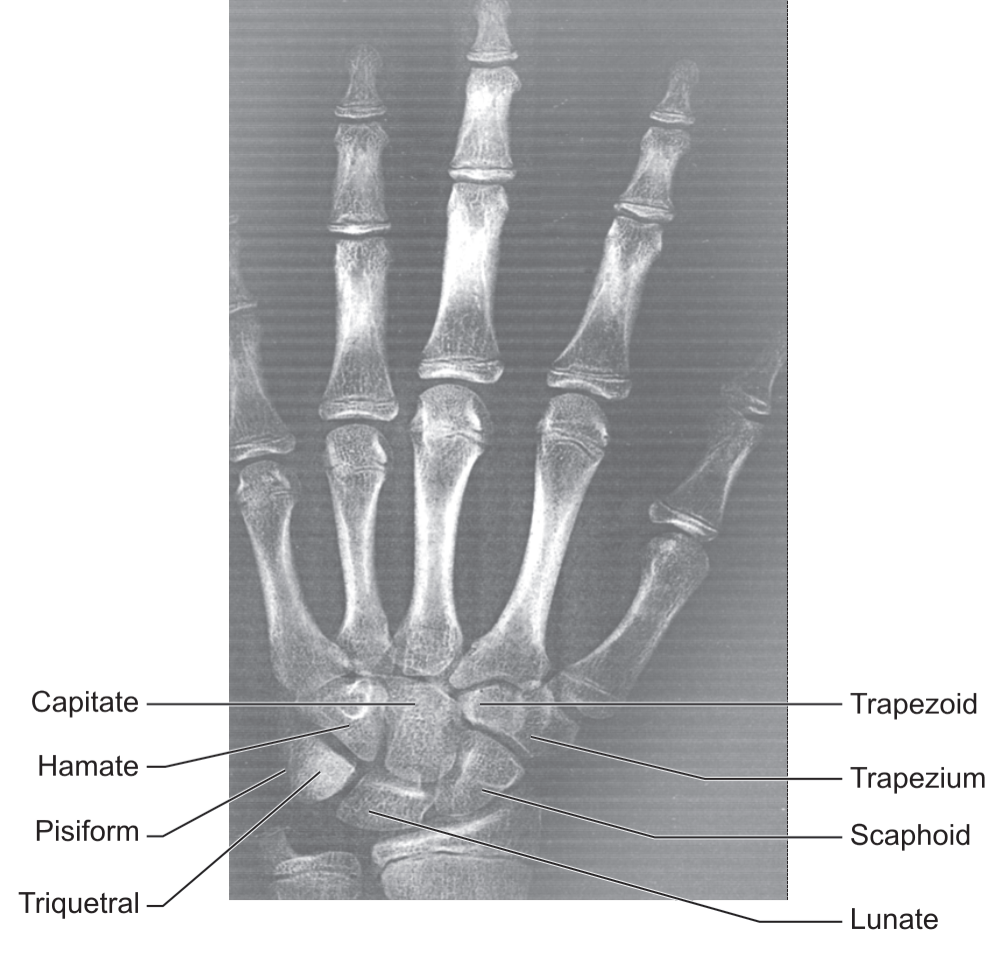
Hand and wrist radiograph showing carpal bones

The skeletal age by hand-wrist radiograph was assessed by evaluating the stages of ossification of various carpal bones (Fig. 2). Bones were then analyzed following a standardized sequence: Capitate, Hamate, Triquetral, Lunate, Scaphoid, Trapezium, Trapezoid, Pisiform. Skeletal age was calculated from standards given to each bone according to the criteria given in “Radiographic atlas” of Greulich and Pyle.^4^ Each hand and wrist radiograph was compared with the atlas pattern of similar age and sex.

**Graph I:**
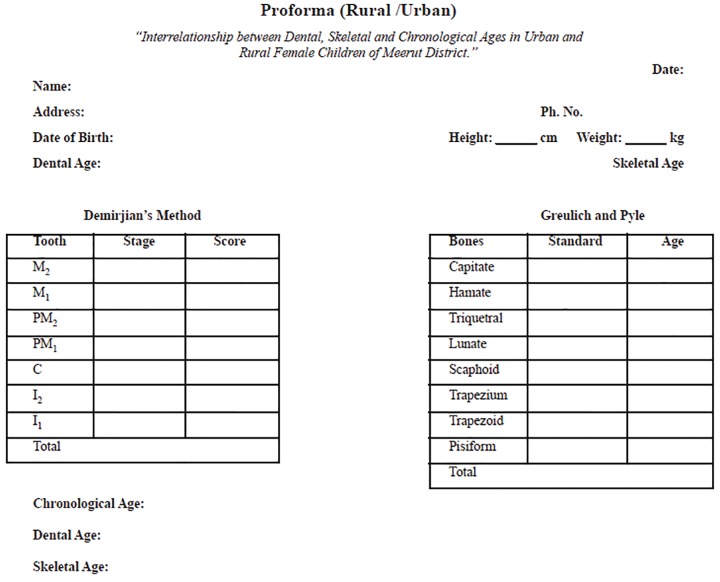
Interrelationship between DA, SA and CA in each group

Finally the data collected was statistically analyzed.

## STATISTICAL ANALYSIS

Data was collected and divided in 4 groups for statistical analysis.

Group I: Rural 8 to 11 years

Group II: Rural 11 to 14 years

Group III: Urban 8 to 11 years

Group IV: Urban 11 to 14 years

Data was statistically analyzed. The descriptive data that included mean, standard deviation were found for each group and used for correlation. Pearson’s correlation coefficient was used to correlate the dental, skeletal and chronological ages of 4 groups. Normal curve test was used to found the difference of these correlations between rural and urban girls. Standard normal variate test (Z-test) was used to find the difference of chronological, dental and skeletal ages between rural and urban female children. The statistical analysis was done by using SPSS version 10.0 software.

For all tests p-value of < 0.05 or < 0.01 was considered for statistical significance.

## RESULTS

### Interrelationship among Dental, Skeletal and Chronological Ages

The overall correlations between dental and skeletal age, chronological age and dental age and between chronological and skeletal age in all groups were correlated by using Pearson’s correlation coefficient shown in Table 3.

The interrelationship among dental, skeletal, and chronological ages in total sample was significant at 0.01 levels.

The following correlation coefficients were found.

### Overall Interrelationship among Dental, Skeletal, and Chronological Ages (Table 3)

A highly significant correlation was found between dental and skeletal ages.

r = 0.752, p < 0.01 (Graph-II)

A strong correlation was also found between chronological and dental ages.

r = 0.650, p < 0.01 (Graph-III)

And between chronological and skeletal ages.

r = 0.620, p < 0.01 (Graph-IV)

The overall interrelationship between the three measures was observed and concluded that interrelationship was better between dental age and skeletal age, than for chronological age-dental age and chronological-skeletal age.

In each group, interrelationship is shown in Graph 1 (Tables 2 to 5). Maximum correlation was found between dental and skeletal ages even in individual groups also.

**Table 3:** **Table 3:** Overall interrelationship among dental, skeletal and chronological ages

					*Chronological* * age*		*Dental age* *(DA)*		*Skeletal* *age*	
	Chronological Age (CA)		Pearson’s correlation (r)		1		0.650		0.620	
			sig. (2-tailed)				0.000		0.000	
			n		80		80		80	
	Dental Age (DA)		Pearson’s correlation (r)		0.650		1		0.752	
			sig. (2-tailed)		0.000				0.000	
			n		80		80		80	
	Skeletal Age (SA)		Pearson’s correlation (r)		0.620		0.752		1	
			sig. (2-tailed)		0.000		0.000			
			n		80		80		80	

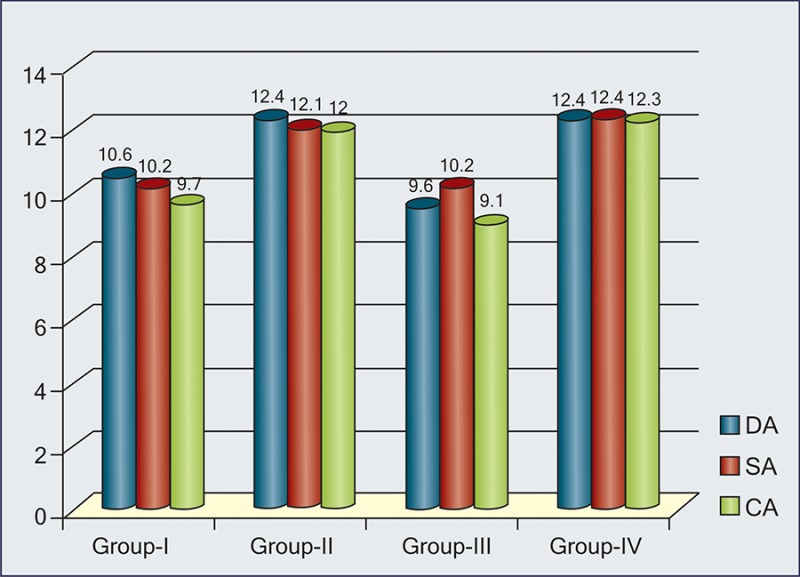



**Graph II:**
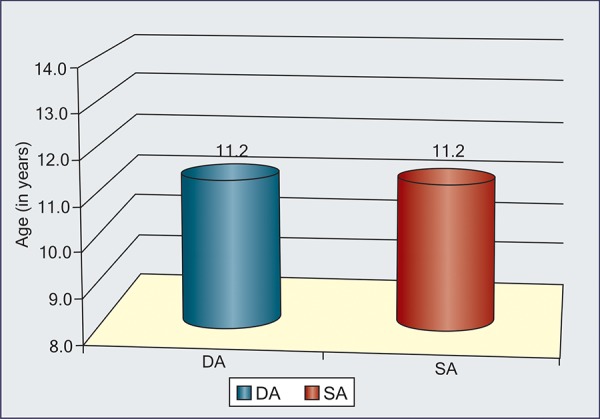
Overall relationship between DA and SA

**Graph III:**
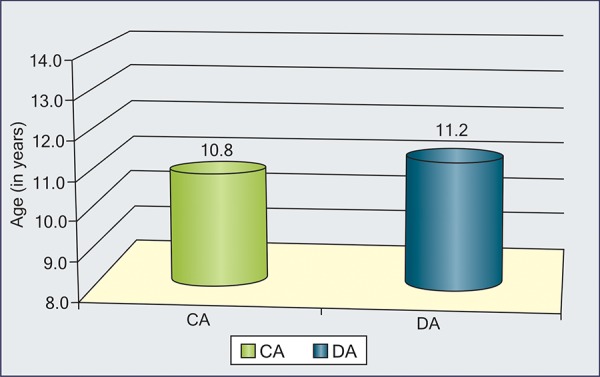
Overall relationship between CA and DA

### Difference in the Correlation Coefficients between Rural and Urban Females

On application of normal curve test at 5% and 1% level of significance, no significant difference was found, i.e. p-value > 0.05 (Table 4) (Graph-V, VI).

**Graph IV:**
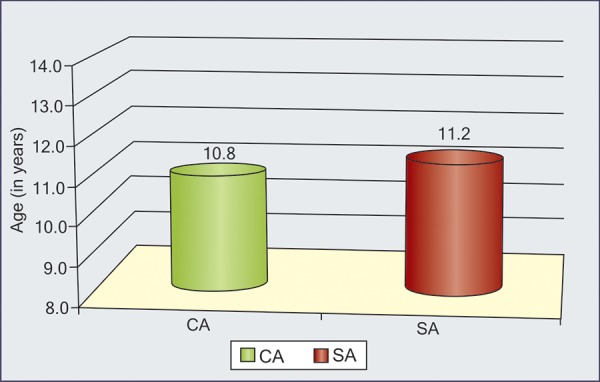
Overall relationship between CA and SA

Hence the inference drawn is that there is no significant difference in interrelationships amongst dental, skeletal, and chronological ages between rural and urban females.

## DISCUSSION

Chronological age, skeletal maturation, dental maturation, secondary sexual characteristics, body weight and height, all constitute an individual’s maturation indices and are used to identify stages of growth. These can be applied separately or together to assess the degree of physiological maturity of a growing child.^6^

It has been shown that chronological age per se is of minimal or no importance and does not constitute a critical factor for estimating the maturation and development of an individual.

Dental maturation is considered as a good index for estimating chronological age.^7^ The relationship between skeletal and physical maturation is well-documented, whereas contradictory views exist concerning their respective relationships with dental age.^8-10^

**Table 4:** **Table 4:** Significance of difference between two correlation coefficients

					*Correlation* *Coefficients*								
	*Age*		*Correlations*		*(r)*				*Z-test*		*p-value*		*Inference*	
					*Rural*		*Urban*							
	8-11 years		DA and SA		0.672		0.535		0.2752		p > 0.05		NS	
			CA and SA		0.344		0.466		0.1851		p > 0.05		NS	
			CA and DA		0.480		0.362		0.1822		p > 0.05		NS	
	11-14 years		DA and SA		0.646		0.571		0.1513		p > 0.05		NS	
			CA and SA		0.549		0.553		0.007		p > 0.05		NS	
			CA and DA		0.483		0.366		0.2953		p > 0.05		NS	

**Graph V:**
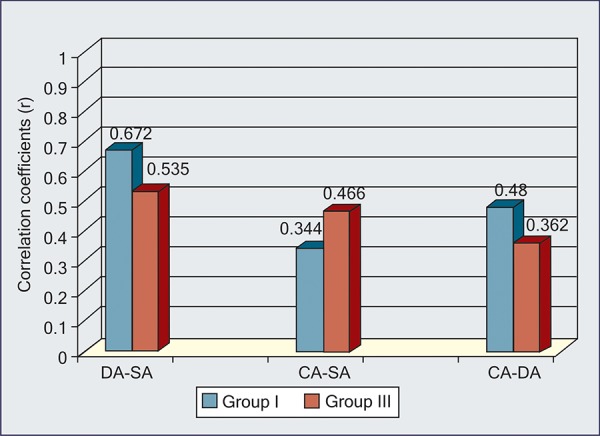
Difference in correlation coefficients between group I and III

**Graph VI:**
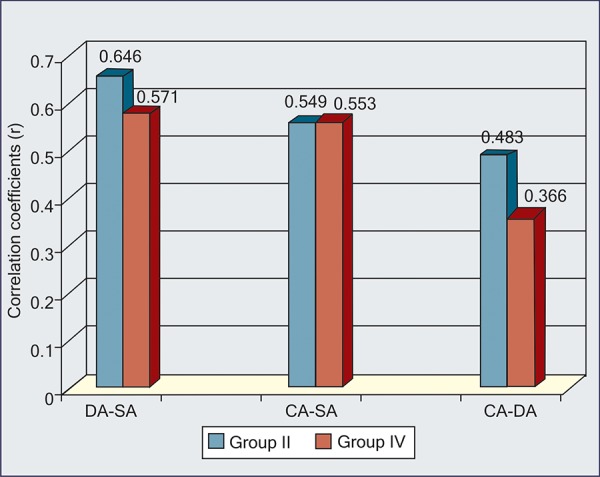
Difference in correlation coefficients between group II and IV

The present study was undertaken to find out the interrelationship amongst dental, skeletal and chronological ages and their difference between rural and urban female children of Meerut district.

Children from urban and rural areas of Meerut district who had no growth disorder were taken to avoid any irregularity in the results, as abnormal or delayed growth can have a significant effect on the dental as well as the skeletal age as stated by Gulati et al.^11^ F Mauricio^12^ also found a significant difference for skeletal maturity and nutrituional status.

Gulati et al^11^ stated that malnutrition can have an adverse effect on the dental as well as on skeletal maturation.

In the present study, highest correlation was observed between the calculated dental age and skeletal age (p < 0.01, r = 0.752). Findings of this study were also in agreement to those of Demisch and Wartmann (1956)^13^ where, r = 0.83, Lee Marjorie MC et al (1963)^14^ (r = 0.85), Liliquist and Lundberg (1971)^15^ (r = 0.88), Engstrom C et al (1983)^16^ (r = 0.88) and Sierra AM (1987)^17^ (r = 0.82). A Gulati (1990)^11^ (r = 0.9594), Prabhakar AR, Panda AK, and Raju OS (2002)^18^ (r = 0.89), Krogman W (2002)^19^ (r = 0.310.69) showed high correlations between skeletal and dental maturity. On the contrary, Sahin-Saglam and Gazilerli (2002)^10^ reported low correlation of dental and skeletal maturation. However, Lewis and Garn (1959)^20^ (r = 0.30), Krogman W (1968)^19^ (r = 0.46) and Demirjian A (1985)^8^ (r = 0.17) have also reported low correlation between dental and skeletal ages.

Correlations between dental and skeletal ages suggest that determination of skeletal maturation is of lesser clinical importance in the treatment planning of children with normal development. The assessment of dental age would suffice in deciding the time of treatment.

The relationship of dental age and chronological age was also statistically significant (p < 0.01, r = 0.650). These findings were also established by Engstrom et al (1983)^16^ (r = 0.77), Demirjian A (1985)^8^ (r = 0.77), Hagg and Matsson (1985)^7^ (r = 0.7-0.9) and Sierra AM (1987)^17^ (r = 0.88), A Gulati (1990)^11^ (r = 0.8635), Prabhakar AR, Panda AK, and Raju OS (2002)^18^ (r = 0.95), Hegde RJ, Sood (2002)^21^ (r = 0.988).

Strong correlation between skeletal age and chronological age was also observed (r = 0.620), Sierra AM (1987)^17^ (r = 0.78), A Gulati (1990)^11^ (r = 0.8716) showed similar results.

Out of the three relationships considered, dental age and skeletal age had the maximum correlations thus indicating that chronological age was comparatively a less reliable index to establish a child’s state of dental and skeletal development. This was also established by A Gulati (1990),^11^ and Prabhakar AR, Panda AK, and Raju OS (2002).^18^ This finding suggest that we do not have to take hand-wrist X-ray for the determination of growth status of a child, OPG would suffice and we can evaluate the growth status by dental age only.

No significant difference of ages and their correlations was found in between urban and rural females. Nowadays not much differences are remaining between rural and urban areas, that’s why difference was not found between two populations.

There is an individual variation of the biologic development in children of similar chronologic age. The goal of methods for age estimation must be to eliminate the meth-odologic variations as much as possible leaving only the unavoidable individual variability. The normal difference between chronologic age and dental age in this study is caused by biologic variation.

An additional explanation may be that the present sample was relatively small and not fully comparable to the material in the reference study. No two individuals grow and develop at the same rate. Nystrom et al^4^ suggested that differences in overall dental maturity exist not only between nations but also between groups of children in a nation with a relatively homogeneous population which can also be seen in this study.
